# Notch3 regulates ferroptosis via ROS‐induced lipid peroxidation in NSCLC cells

**DOI:** 10.1002/2211-5463.13393

**Published:** 2022-03-18

**Authors:** Zhikang Li, JinYang Xiao, Mengyu Liu, Jiaqi Cui, Bowen Lian, Yuanlu Sun, Chunyan Li

**Affiliations:** ^1^ Department of Pharmaceutical Science China Medical University‐The Queen's University of Belfast Joint College Shenyang China; ^2^ School of Life Sciences China Medical University Shenyang China; ^3^ Science Experiment Center China Medical University Shenyang China

**Keywords:** ferroptosis, GPX4, Notch3, NSCLC, PRDX6, ROS

## Abstract

Ferroptosis is type of programmed cell death, which is known to be involved in certain cancers. Notch3 signaling is reported to be involved in the tumorigenesis of non‐small‐cell lung cancer (NSCLC) and regulates iron metabolism, lipid synthesis, and oxidative stress in some tissues. However, whether Notch3 signaling regulates ferroptosis is unclear. In this study, we found that ferroptosis inhibitors, ferrostatin‐1 and liproxstatin‐1, protected against cell death induced by Notch3 knockdown and that Notch3 knockdown initiated ferroptosis in NSCLC cells by increasing reactive oxygen species (ROS) levels, lipid peroxidation, and Fe^2+^ levels, accompanied by downregulation of glutathione peroxidase 4 (GPX4) and peroxiredoxin6 (PRDX6). Conversely, Notch3 intracellular domain overexpression suppressed erastin‐induced ferroptosis, which was synergistically enhanced by MJ33 in H1299 cells via a decrease in ROS levels and lipid peroxidation, accompanied by upregulation of GPX4 and PRDX6. Moreover, Notch3 knockdown decreased tumorigenesis *in vivo* with downregulation of GPX4 and PRDX6. In summary, here we have identified Notch3 as a potential negative regulator of ferroptosis in NSCLC.

## Graphical abstract

Notch3 acts as a potential negative regulator of ferroptosis in non‐small‐cell lung cancer. Notch3 knockdown induced ferroptosis as well as downregulation of GPX4 and PRDX 6. Conversely, Notch3 intracellular domain suppresses erastin‐induced ferroptosis synergistically with MJ33. Taken together, our findings provide new insights into a potential strategy for cancer therapy based on targeting Notch3 and ferroptosis.

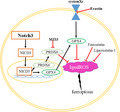

**Abbreviations**
GPX4: glutathione peroxidase 4
IHC: immunohistochemistry
iPLA2: independent phospholipase A2
MDA: malondialdehyde
MFI: mean fluorescence intensity
NICD3: Notch3 intracellular domain
NSCLC: non‐small‐cell lung cancer
PCD: programmed cell death
PRDX6: peroxiredoxin 6
ROS: reactive oxygen species
SCLC: small‐cell lung cancer
SEM: standard error of mean

Lung cancer remains the leading cause of cancer mortality worldwide, including two main types, small‐cell lung cancer (SCLC) and non‐small‐cell lung cancer (NSCLC) [1, 2]. Compared with SCLC, NSCLC is resistant to chemotherapy and radiotherapy. Therefore, understanding the molecular mechanisms that drive NSCLC may facilitate development of novel diagnostic biomarkers and targeted therapies.

Programmed cell death (PCD) is crucial to maintain tissue homeostasis and development. Dysfunctional PCD leads to various diseases such as cancer. Triggering PCD by anticancer drugs is a therapeutic approach to kill cancer cells [3]. However, the effectiveness of this approach is presently suboptimal because of the limited understanding of the complex mechanisms that underlie PCD [4].

Ferroptosis is a newly recognized form of PCD, which is distinct from other forms of PCD, such as apoptosis, autophagy, and programmed necrosis. Ferroptotic cells have smaller mitochondria with decreased cristae and an increased membrane density, but not any hallmarks of apoptosis. Moreover, inhibiting apoptosis, autophagy, and programmed necrosis does not protect cells from ferroptosis. Mechanistically, to date, accumulation of iron and reactive oxygen species (ROS) inside cells and lipid peroxidation are considered as the hallmarks and cause of ferroptosis. In many cancers, inducing ferroptosis exerts a significant suppressive effect. Therefore, ferroptosis provides new therapeutic strategies for killing cancer cells [5].

Because iron and ROS inside cells and lipid peroxidation are involved in ferroptosis, the genes and signaling pathways which regulate iron metabolism, lipid synthesis, and oxidative stress could potentially modulate sensitivity to ferroptosis. The Notch signaling pathway is important for tissue development and homeostasis, and its dysregulation promotes cancerogenesis in many cell types. Increasing diffuse iron accumulation has been observed in the putamen and caudate nucleus of patients with a Notch3 mutation [6]. Mice fed an iron‐deficient diet had significantly higher tumor volumes and lung metastasis because of a Notch alteration [7]. In lung alveolar epithelial type II cells and SK‐N‐MC cells, Notch regulates lipid peroxidation and intracellular ROS levels [8, 9]. In the liver, the Notch signaling pathway and lipid synthesis are closely related to liver fat accumulation [10]. Resveratrol decreases lipid peroxidation by inhibition of Notch signaling in CCl₄‐induced liver injuries [11]. Because that these evidence suggest the critical roles of the Notch signaling pathway in regulating the levels of iron, ROS, and lipid peroxidation, it is of particular importance to determine whether Notch3 signaling regulates ferroptosis, which has been rarely reported.

In the present study, we analyzed the relationship between Notch3 expression and ferroptosis. We found that specially inhibiting Notch3 induced ferroptosis with downregulation of glutathione peroxidase 4 (GPX4) and peroxiredoxin 6 (PRDX6), whereas overexpressing Notch3 intracellular domain (NICD3) suppressed ferroptosis induced by erastin, which was synergistically enhanced by MJ33 with the upregulation of GPX4 and PRDX6. Consistent with the *in vitro* results, specially inhibiting Notch3 decreased tumorigenesis with downregulation of GPX4 and PRDX6 in a xenograft mouse model. Thus, Notch3 may be a novel regulator of ferroptosis, which provides new insight into targeting Notch3 as a promising therapeutic option in NSCLC.

## Methods

### Cell culture and transfection

The human NSCLC cell lines H1299, H460, and A549 were originally purchased from National Collection of Authenticated Cell Cultures (Baltimore, MD, USA) and propagated in conventional FBS‐supplemented RPMI1640 and DMEM/F‐12K (Gibco, Carlsbad, CA, USA), respectively. For specially inhibiting Notch3, the cells were transfected with Notch3 shRNA lentiviral particles (sc‐37136‐V; Santa Cruz, Dallas, TX, USA) and stable cell lines were established (described previously [12]). For overexpression of NICD3, a pCMV14‐Nero vector containing the coding sequence of NICD3 was transfected into cells and stable cell lines were established (described previously [13]). The transfection efficiency was confirmed by western blot analysis.

### Induction and inhibition of ferroptosis

Erastin, a ferroptosis inducer, was purchased from Selleck Biological Corporation (S7242; Shanghai, China). 1‐Hexadecyl‐3‐(trifluoroethyl)‐sn‐glycero‐2‐phosphomethanol lithium (MJ33), an independent phospholipase A2 (iPLA2) activity inhibitor, was purchased from Sigma‐Aldrich (M3315‐5MG; St. Louis, MO, USA). Ferrostatin‐1 and liproxstatin‐1, the ferroptosis inhibitors, were purchased from MCE Biological Corporation (HY‐100579 and HY‐12726; Shanghai, China). In the present study, the final working concentrations of erastin, MJ33, ferrostatin‐1, and liproxstatin‐1 were 15, 5, 1, and 10 μm, respectively.

### Cell viability assay

Cells were resuspended in 96‐well plates at 10^4^ cells·mL^−1^ concentration. After cultured for 24 h or 48 h, cell viability was checked by Cell Counting Kit‐8 (CCK‐8; Beyotime Co, Shanghai, China).

### ROS assay

Cells were incubated in serum‐free media with 10 μm 2′,7′‐dichlorodihydrofluorescein diacetate (H2DCF‐DA; S0033S; ROS Assay Kit; Beyotime Co.) for 20 min. Then, the cells were washed three times with PBS to terminate the reaction. Following, cells were trypsinized and resuspended in PBS. The mean fluorescence intensity (MFI) of the cells was measured using flow cytometer (FACSCalibur; BD Biosciences, San Jose, CA, USA).

### Lipid peroxidation assay

Cells were trypsinized, washed with ice‐cold PBS three times, and lysed in cell lysis buffer. Then, the lysate was centrifuged at 10,000 ***g*** for 10 min. To evaluate lipid peroxidation, the concentration of malondialdehyde (MDA) in the supernatant was examined using a lipid peroxidation MDA assay kit (S0131S; Beyotime Co.).

### Fe^2+^ intensity assay

Cells was collected and washed with cold PBS buffer. Then, cells were incubated with Phen green SK (PGSK; Thermo Fisher Scientific, Waltham, MA, USA) at 10 µm at 37 °C for 30 min in dark. Then, cells were collected, washed, and analyzed by flow cytometry (LSRFortessa; BD Biosciences) within 1 h. No fluorescent probe samples served as the negative control.

### Western blot analysis

Cells were lysed in RIPA lysis buffer (P0013C; Beyotime Co.). Cell extracts were collected by centrifugation, analyzed by 12% or 10% SDS/PAGE, and then followed by western blotting using mouse monoclonal anti‐GPX4 (67763‐1‐Ig; ProteinTech Group, Wuhan, China), rabbit polyclonal anti‐Notch3 (ab23426; Abcam, Cambridge, UK), and rabbit polyclonal anti‐PRDX6(13585‐1‐AP; ProteinTech Group). After the primary incubation, membranes were incubated with secondary horseradish peroxidase (HRP)‐conjugated goat anti‐rabbit or anti‐mouse IgG (A0279 or A0288; Beyotime Co.). Protein bands were visualized using SuperSignal West Pico Chemiluminescent Substrate (Thermo Fisher Scientific, Waltham, MA, USA). The integrated optical density of band was analyzed using imagej software (National institutes of health, Bethesda, MD, USA).

### Xenograft animal model

Six‐week‐old BALB/c nude male mice were purchased from HFK Bio (Beijing, China). All experimental protocols and procedures were approved by the animal ethics committee of China Medical University (CMU2021288), and all experiments were performed in compliance with the instructions of Laboratory Animals Center, China Medical University. A 14 : 10 h light/dark cycle was used, and all mice had free access to food and water. Food was replenished, and the cage was cleaned once a week. Cells (1 × 10^7^ tumor cells/100 μL PBS) were subcutaneously injected into the right lower flank of BALB/c nude mice. The tumor growth was monitored every 2–3 days. At the end of the experiment, the animals were sacrificed with cervical dislocation, and the tumors were separated from the surrounding muscles and dermis. Then, the tumors were weighed. Moreover, the tumor volumes were measured with Vernier calipers and calculated by the following formula: (length × width^2^)/2.

### Immunohistochemistry

Tumor tissues were fixed with 4% paraformaldehyde solution for 24 h, paraffin‐embedded, and sectioned at 4 μm. The sections were deparaffinized, rehydrated, and subjected to an antigen retrieval treatment with Tris/EDTA buffer (pH 9.0) at 100 °C for 25 min. The sections were incubated in 3% H_2_O_2_ for 30 min to quench endogenous peroxidase activity. The sections were blocked with goat serum (ZSGB Bio, Beijing, China) for 1 h and then incubated with mouse monoclonal anti‐GPX4 (67763‐1‐Ig; ProteinTech Group), rabbit polyclonal anti‐Notch3 (ab23426; Abcam), and rabbit polyclonal anti‐PRDX6 (13585‐1‐AP; ProteinTech Group) overnight at 4 °C, respectively. Next day, the slides were washed three times for10 min in PBS and incubated in biotinylated anti‐mouse or rabbit secondary antibody at room temperature for 2 h (ZSGB Bio). The peroxidase reaction was developed with diaminobenzidine and peroxide. Finally, the sections were counterstained with hematoxylin and mounted in crystal mount medium. The expressions of Notch3, GPX4 and PRDX6 were evaluated on a light microscope (IX51; Olympus, Tokyo, Japan).

### Statistical analysis

All experiments were performed in the triplicate, and data are presented as the mean ± standard error of mean (SEM). Statistical analysis was performed using graphpad prism 6.0 (GraphPad Software Inc., San Diego, CA, USA). Two‐way ANOVA was utilized for multiple comparison. The differences between two groups were analyzed using two‐tailed Student's *t* tests. *P* < 0.05 was considered to indicate statistically significant differences (*P < 0.05 and **P < 0.01).

## Results

### Specific inhibition of Notch3 induces ferroptosis in NSCLC cells

**Fig. 1:**
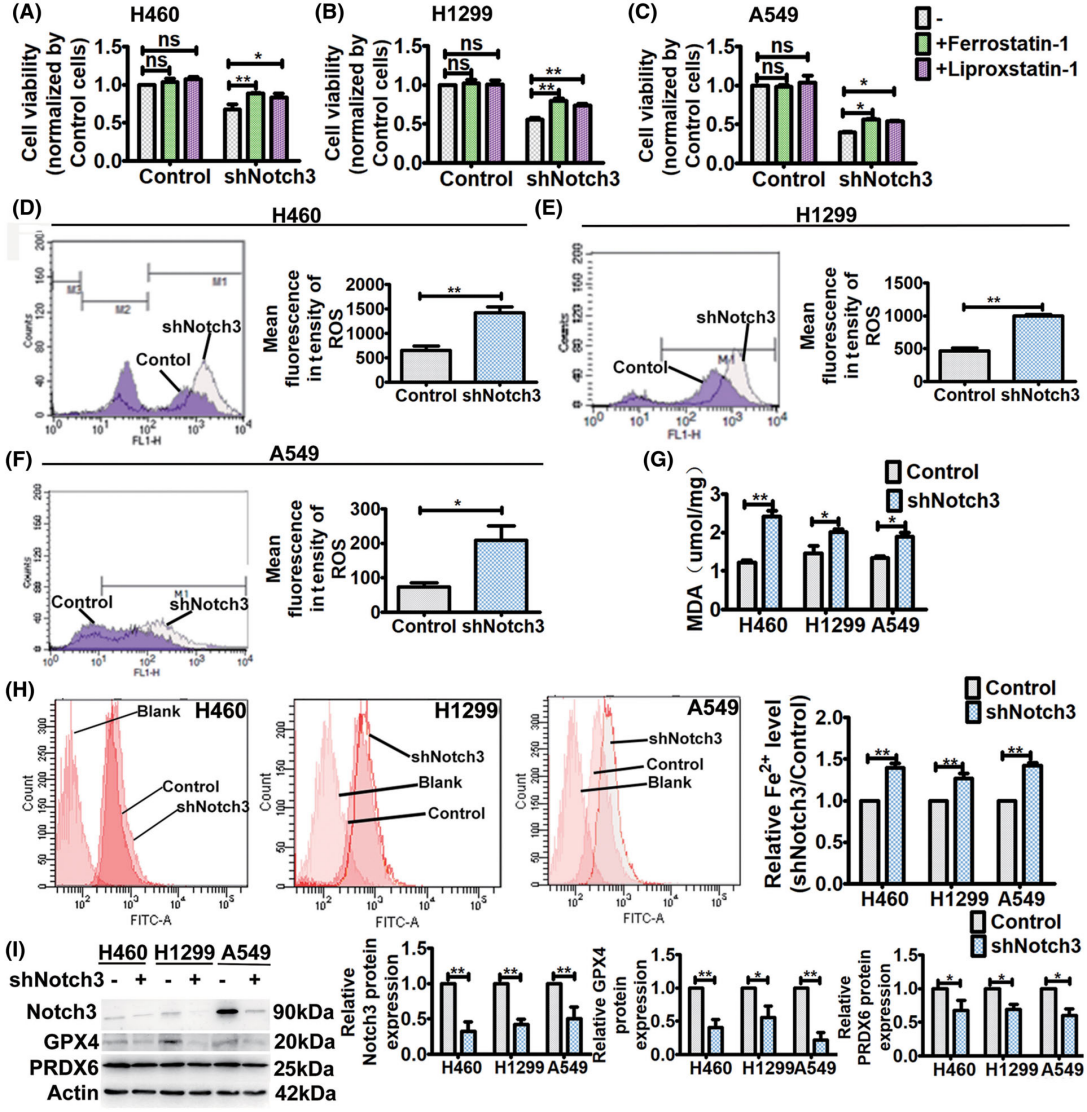
Notch3 knockdown induces ferroptosis. (A–C) After ferrostatin‐1 (1 μm) and liproxstatin‐1 (10 μm) treatment for 48 h, respectively, cell viability was assessed by CCK8 assays. Data were reported as mean ± SEM for three independent experiments, and statistical analysis was performed via two‐way ANOVA. (D–F) ROS levels in control and Notch3 knockdown cells were measured by flow cytometry using H2DCF‐DA. Left, representative flow cytometry data. Right, histogram of MFI of ROS. Data were reported as mean ± SEM for three independent experiments, and statistical analysis was performed via two‐tailed Student's *t* tests. (G) Lipid peroxidation in control and Notch3 knockdown cells was assessed by MDA assay. Data were reported as mean ± SEM for three independent experiments, and statistical analysis was performed via two‐way ANOVA. (H) Fe^2+^ levels in control and Notch3 knockdown cells were assayed by flow cytometry using PGSK. Left, representative flow cytometry data. Right, histogram of the relative Fe^2+^ level. Data were reported as mean ± SEM for three independent experiments, and statistical analysis was performed via two‐way ANOVA. (I) Western blot analysis of Notch3, GPX4, and PRDX6 expressions in control and Notch3 knockdown cells. Left, representative western blot. Right, histogram of protein expressions. Data were reported as mean ± SEM for three independent experiments, and statistical analysis was performed via two‐way ANOVA; *P < 0.05 and **P < 0.01.

In our previous study, we found that specific inhibition of Notch3 with Notch3 shRNA killed NSCLC cells and inhibited the cell proliferation [12]. To determine whether ferroptosis is a form of the Notch3 shRNA‐induced cell death, we treated cells with ferroptosis inhibitors, ferrostatin‐1 and liproxstatin‐1, respectively. The treatment for 48 h affected cell viability of Notch3 knockdown cells, but not that of the control cells (Fig. 1A–C). We observed fewer dead Notch3 knockdown cells treated with the ferroptosis inhibitors compared with untreated cells, which indicated that ferroptosis contributed to Notch3 knockdown‐induced cell death (Fig. 1A–C).

Next, we investigated whether Notch3 knockdown induced ferroptosis. Increased ROS, iron, and lipid peroxidation are markers and cause of ferroptotic cell death. In this study, we found that Notch3 knockdown significantly increased the ROS level inside cells (Fig. 1D–F). In line with this, Notch3 knockdown also significantly increased iron inside cells and lipid peroxidation (Fig. 1G,H). Then, the effects of Notch3 knockdown on the protein expressions of GPX4 and PRDX6 were investigated. GPX4 functions as a negative regulator of ferroptosis by decreasing ROS production [14]. As a dual‐negative regulators of ferroptosis, beside GPX activity, PRDX6 removes lipid ROS through its iPLA2 activities [15]. As shown in Fig. 1I, Notch3 knockdown significantly decreased the expressions of GPX4 and PRDX6 in NSCLC cells. Taken together, these findings indicate that specific inhibition of Notch3 initiated ferroptosis by increasing ROS induced lipid peroxidation through GPX4 and PRDX6.

### Stable NICD3 overexpression suppresses ferroptosis induced by erastin, which is synergistically enhanced by MJ33 in H1299 cells

**Fig. 2:**
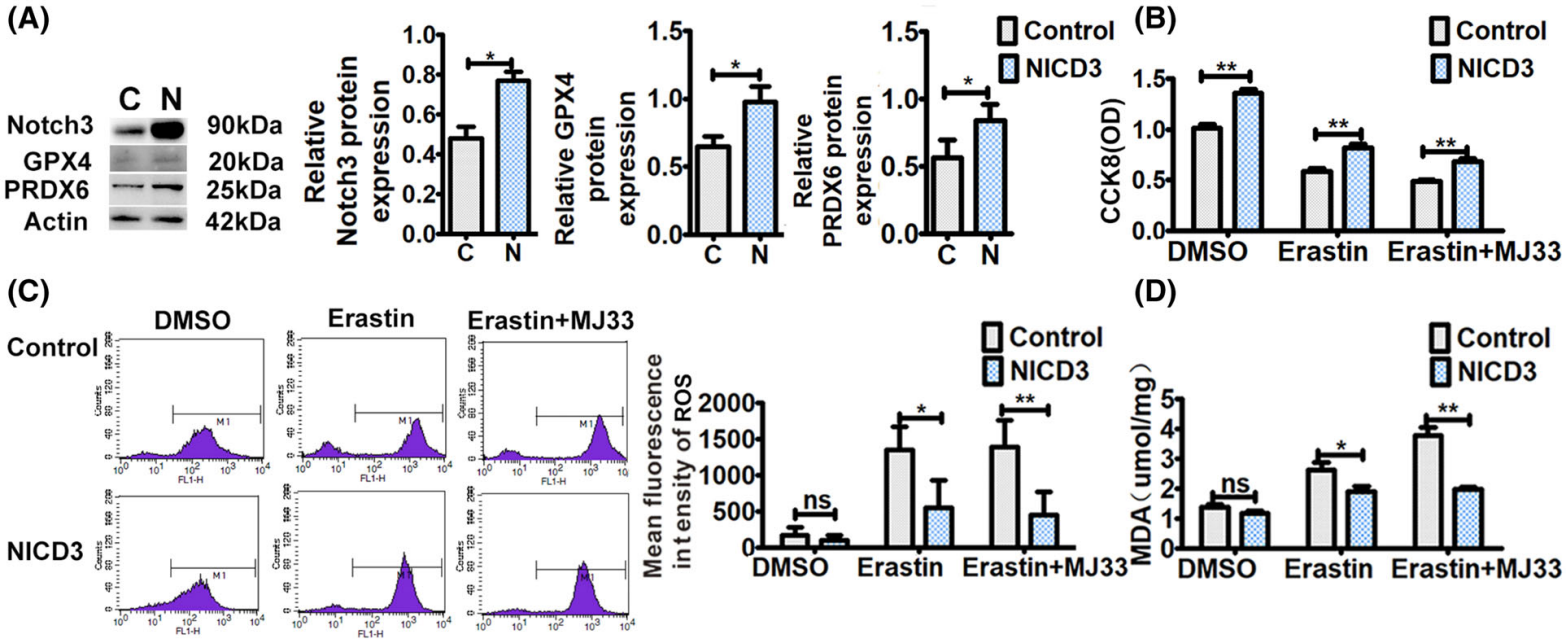
NICD3 overexpression suppresses ferroptosis. (A) Western blot analysis of Notch3, GPX4, and PRDX6 expressions after transfecting an NICD3 sequence‐containing plasmid. Left, representative western blot. Right, histogram of protein expressions. Data were reported as mean ± SEM for three independent experiments, and statistical analysis was performed via two‐tailed Student's *t* tests. (B) After treatment with or without erastin (15 μm) and/or MJ33 (5 μm) for 24 h, cell viability was assessed by CCK8 assays. Data were reported as mean ± SEM for three independent experiments, and statistical analysis was performed via two‐way ANOVA. (C) After treatment with or without erastin (15 μm) and/or MJ33 (5 μm) for 24 h, ROS levels in cells were measured by flow cytometry using H2DCF‐DA. Left, representative flow cytometry data. Right, histogram of MFI of ROS. Data were reported as mean ± SEM for three independent experiments, and statistical analysis was performed via two‐way ANOVA. (D) After treatment with or without erastin (15 μm) and/or MJ33 (5 μm) for 24 h, lipid peroxidation in cells was assessed by MDA assays. Data were reported as mean ± SEM for three independent experiments, and statistical analysis was performed via two‐way ANOVA; *P < 0.05 and **P < 0.01.

We found that Notch3 knockdown induced ferroptosis. However, whether Notch3 regulated ferroptosis under ferroptotic stress remained unknown. Erastin inhibits cystine‐glutamate transport receptor, which reduces GPX4 activity [16]. Subsequently, the cellular antioxidant capacity reduces, thereby increasing lipid peroxidation and finally leading to ferroptosis. Therefore, erastin has been considered to be an initial factor of ferroptosis. In this study, an NICD3 sequence‐containing plasmid was stably transfected into H1299 cells, which are deficient in p53, and the effects of NICD3 overexpression on erastin‐induced ferroptosis were observed. After the transfection, the expressions of NICD3, GPX4, and PRDX6 were upregulated in H1299 cells (Fig. 2A). As shown in Fig. 2B, overexpression of NICD3 significantly abolished erastin‐induced growth inhibition of H1299 cells. In parallel with the cell growth pattern, overexpression of NICD3 also inhibited both erastin‐induced ROS and MDA in H1299 cells (Fig. 2C,D). These data implied that NICD3 overexpression suppressed erastin‐induced ferroptosis by decreasing ROS and lipid peroxidation through GPX4 and PRDX6.

PRDX6 has both GPX and iPLA2 activities. MJ33, a specific iPLA2 inhibitor, has been reported to enhance erastin‐induced ferroptosis by limiting the removing of lipid ROS [15]. Similar to this report, compared with erastin treatment, blocking the iPLA2 activity using MJ33 combined with erastin significantly increased erastin‐induced cellular ROS and MDA levels in H1299 cells (Fig. 2C,D), which suggested that MJ33 promoted ferroptotic stress initiated by erastin. However, the ROS and MDA levels increased by the combination of MJ33 and erastin were still reduced by overexpression of NICD3. These data suggested that NICD3 also suppressed MJ33‐enhanced ferroptotic stress in H1299 cells by decreasing lipid ROS through recovering the iPLA2 activity.

### Specific inhibition of Notch3 decreases tumorigenesis in BALB/C nude mice with downregulation of GPX4 and PRDX6

**Fig. 3:**
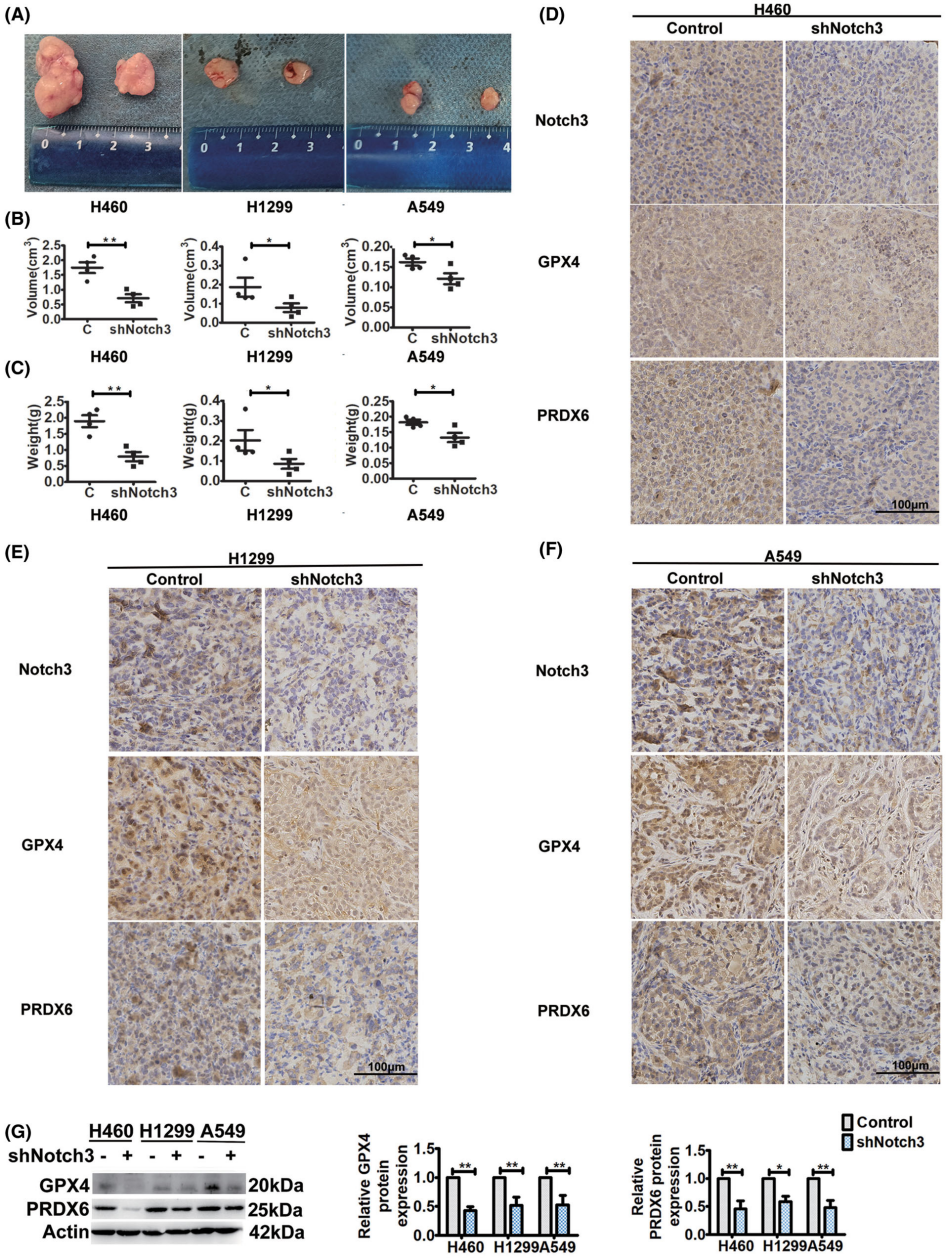
Notch3 knockdown decreases tumorigenesis with downregulation of GPX4 and PRDX6 *in vivo*. (A) Representative xenografted tumor images. (B) Volumes of tumors formed in BALB/c athymic nude mice. Data were reported as mean ± SEM, and statistical analysis was performed via two‐tailed Student's *t* tests. (C) Weights of tumors formed in BALB/c athymic nude mice. Data were reported as mean ± SEM, and statistical analysis was performed via two‐tailed Student's *t* tests. (D–F) Representative immunohistochemical staining of Notch3, GPX4, and PRDX6. All sections were counter‐stained with hematoxylin. Scale bar, 100 μm. (G) Western blot analysis of GPX4 and PRDX6 expressions of tumors. Left, representative western blot. Right, histogram of protein expressions. Data were reported as mean ± SEM, and statistical analysis was performed via two‐way ANOVA; *P < 0.05 and **P < 0.01.

*In vitro* experiments, we showed that specific inhibition of Notch3 decreased the viability of NSCLC cells with downregulation of GPX4 and PRDX6. To verify the roles of Notch3 in tumorigenesis and the relationship of Notch3 with GPX4 and PRDX6, we established an *in vivo* xenograft model using Notch3 knockdown lung cancer cells. At 14 days after injecting the cells, all mice were sacrificed, tumors were dissected out, and their volume and weight were measured. As shown in Fig. 3A–C, there were significant differences in tumor volumes and weights between the control and the Notch3 knockdown mice. Similar to the *in vitro* results, Notch3 knockdown effectively decreased the tumor volumes and weights. In tumors of Notch3 knockdown mice, immunohistochemistry (IHC) analysis revealed significant downregulation of GPX4 and PRDX6 compared with the control (Fig. 3D–F), which was confirmed by western blot analysis (Fig. 3G). Overall, these findings indicated that specific inhibition of Notch3 decreased tumorigenesis in BALB/C nude mice, which was closely related to downregulation of GPX4 and PRDX6.

## Discussion

As an evolutionarily conserved cell–cell communication pathway, Notch signaling, particularly Notch1 and Notch3, plays major roles in lung development and disease [17]. Upregulation of Notch1/3 signaling has been linked to the initiation and progression of lung cancer [17, 18, 19]. In our previous studies, we found that Notch3 is an oncogene in NSCLC and inhibiting Notch3 decreases cell proliferation [12], but the molecular mechanisms of Notch3 involvement in NSCLC are not well characterized.

Ferroptosis has recently been considered to be a type of cell death distinct from apoptosis, one that also eliminates cancer cells [5]. In the present study, we attempted to understand whether Notch3 signaling regulated ferroptosis in NSCLC. First, under normal conditions, specific inhibition of Notch3 induced ferroptosis in H460, H1299, and A549 cells. Furthermore, NICD3 overexpression suppressed the ferroptotic stress initiated by erastin, which was synergistically enhanced by MJ33 in H1299 cells. Taken together, our data indicate that Notch3 is a regulator of ferroptosis.

Upon binding of a Notch receptor to a ligand located on a neighboring cell, the signaling is initiated. Signaling via Notch receptor, a single‐pass transmembrane protein with extracellular, transmembrane, and intracellular domains, proceed by two successive proteolytic cleavages, leading to accumulation of NICD and its translocation into the nucleus. Then, NICD interacts with a transcription complex that modulates the expression of downstream target genes to exert Notch actions [20, 21]. Although the present study did not provide direct evidence that Notch3 regulated the transcription of GPX4 and PRDX6, we assume that the changes in the expressions of GPX4 and PRDX6 were caused by Notch3 because the expression levels of GPX4 and PRDX6 were changed in accordance with the expression levels of Notch3 *in vitro* and *in vivo*.

As an important mediator of ferroptosis, GPX4 directly reduces phospholipid hydroperoxide [22]. Knockdown of GPX4 induces cell death in embryos, testes, brain, liver, heart, and photoreceptor cells of mice in a lipid peroxidation‐dependent manner. GPX4 inhibits ferroptosis by decreasing phospholipid peroxidation induced by ferroptosis initiators [14, 23]. In human nucleus pulposus cells, heme‐induced ferroptosis with downregulation of GPX4 levels might be associated with the Notch pathway [24]. PRDX6 is a bifunctional protein with GPX and iPLA2 activities, which contributes to tumor necrosis factor‐ or oxidative stress‐induced apoptosis [25, 26]. PRDX6 promotes certain cancers' progression through its GPX and iPLA2 activities [27, 28, 29]. Although knockdown of PRDX6 did not initiate ferroptosis, specific inhibition of iPLA2 cooperatively enhances erastin‐induced ferroptosis [15]. In lung cancer, loss of presenilin 2, which activates Notch signaling, is associated with increased iPLA2 activity and lung tumor development [30]. Therefore, Notch3 may regulate ferroptosis in NSCLC cells at last partially through GPX4 and PRDX6 that modulate ROS inside cells and lipid peroxidation.

Although further studies on the implication of Notch3 in ferroptosis of NSCLC and its possible mechanisms are required, our present data increase our understanding of the Notch3 signaling pathway and suggest that manipulating the Notch3 signaling is a promising therapeutic strategy for NSCLC.

## Conclusion

In conclusion, our *in vitro* data support the hypothesis that Notch3 acts as a negative regulator of ferroptosis in NSCLC. Notch3 knockdown significantly induces ferroptosis, whereas overexpression of NICD3 suppresses the ferroptosis induced by erastin, which is synergistically enhanced by MJ33. Mechanistically, the regulatory effects of Notch3 on ferroptosis are highly related to its regulation of GPX4 and PRDX6 expressions. Moreover, our *in vitro* results were corroborated by the results of *in vivo* experiments performed in the xenograft model, in which Notch3 knockdown decreased tumorigenesis with downregulation of GPX4 and PRDX6. Our findings provide new insights into a potential strategy for cancer therapy by targeting both Notch3 and ferroptosis.

## Conflict of interest

The authors declare no competing interests.

## Author contributions

CL and ZL contributed to the study conception and design. ZL, JX, ML, BL, JC and YS performed material preparation, data collection, and analysis. The first draft of the manuscript was written by CL and ZL, and all authors commented on previous versions of the manuscript. All authors read and approved the final manuscript.

## Data Availability

The data in the present study are available from the corresponding author on reasonable request.
